# Effects of Integrated Sports Vision Training Batteries on Time- and Accuracy-Dependent Outcomes in Sport-Specific Tasks: A Systematic Review and Meta-Analysis

**DOI:** 10.1007/s44402-026-00147-8

**Published:** 2026-07-28

**Authors:** Jesús Vera, Beatriz Redondo, Amador García-Ramos, Mario Cantó-Cerdán

**Affiliations:** 1 CLARO (Clinical and Laboratory Applications of Research in Optometry) Laboratory, Department of Optics, Faculty of Sciences, University of Granada, Granada, Spain; 2 New England College of Optometry, Boston, Massachusetts USA; 3 Department of Physical Education and Sport, Faculty of Sport Sciences, University of Granada, Granada, Spain; 4 Department of Sports Sciences and Physical Conditioning, Faculty of Education, Universidad Católica de la Santísima Concepción, Concepción, Chile; 5 Department of Optometry, Vissum (Miranza Group), Alicante, Spain; 6 Research group in Optometry (GIOptom), University of Alicante, Alicante, Spain

**Keywords:** Perceptual–cognitive training, Performance transfer, Optometry, Sports performance

## Abstract

**Objectives:**

This systematic review and meta-analysis sought to quantify the effects of integrated sports vision training (SVT) batteries on time- and accuracy-dependent outcomes assessed during sport-specific tasks and to examine methodological quality and sources of heterogeneity across studies.

**Methods:**

Following PRISMA 2020 guidelines, PubMed, Embase, Scopus and Web of Science were searched from inception to October 21, 2025. Eligible studies investigated multicomponent visual—cognitive or perceptual—motor training batteries in healthy athletes and reported sport-relevant performance outcomes. Random-effects meta-analyses were conducted separately for time-dependent and accuracy-dependent measures using standardised mean differences (SMDs). Methodological quality was assessed using a modified Downs and Black checklist.

**Results:**

Seventeen studies were included in the systematic review, with 14 contributing to the meta-analysis. Integrated SVT batteries produced large and statistically significant improvements in response time (SMD = −2.67, 95% CI [−3.82, −1.52]) and response accuracy (SMD = −1.18, 95% CI [−1.85, −0.51]) compared with control conditions. Substantial heterogeneity was observed, particularly for time-dependent outcomes (*I*² = 92%). However, sensitivity analyses confirmed the robustness of the pooled effects, with consistent direction and statistical significance across models.

**Conclusions:**

Integrated SVT batteries improve time- and accuracy-dependent outcomes during sport-specific tasks, suggesting potential transfer to sport performance. These findings extend prior work on isolated visual or perceptual–cognitive training by demonstrating that multicomponent, ecologically valid interventions are more likely to promote far transfer to sport performance. Future research should focus on optimising training design, examining long-term retention and identifying moderators of training responsiveness to inform evidence-based practice further.

Key points

- This meta-analysis suggests that training programmes combining vision, decision-making and movement can help athletes respond faster and more accurately during realistic sport tasks.
- Sports vision training may be most useful when it reflects the demands of competition, including meaningful choices, time pressure and coordinated movement.
- Researchers should now focus on which training designs work best, how long benefits last and which athletes are most likely to improve.

## Introduction

Sports performance depends on the efficient integration of perceptual, cognitive and motor systems [1]. Meta-analytic evidence shows that high-level athletes outperform their lower-level counterparts on perceptual–cognitive tasks related to cue pickup, response accuracy and response time, highlighting the importance of perceptual–cognitive efficiency in competitive success [2]. Further, a separate meta-analysis indicated that athletes often show advantages on laboratory cognition tasks, suggesting domain-general benefits that may support sports performance [3]. These findings motivate training approaches that target visual, perceptual–cognitive and visuomotor abilities with the expectation that general gains translate into sport-specific performance.

Sports vision training (SVT), defined as structured interventions designed to enhance visual, perceptual–cognitive and visuomotor abilities to improve sport-specific performance, has gained interest in sport contexts, with recent reviews summarising tools, taxonomies and applied directions [4,5]. Traditional SVT approaches have often adopted a component-based paradigm, focusing on isolated skills such as saccadic control, vergence, reaction time or visual memory. Although these methods may yield short-term improvements in laboratory-based measures, evidence for far transfer to sport performance remains inconsistent [6,7]. In response, contemporary frameworks such as the Modified Perceptual Training Framework [8] and representative learning design [9] emphasise that training should preserve the natural coupling between perception, cognition and action through the incorporation of sport-representative tasks, thereby enhancing ecological validity and the likelihood of transfer. From this perspective, interventions that integrate multiple perceptual–motor components within sport-representative contexts may be better suited to facilitate transfer to real-world performance [10].

The strongest evidence for performance improvements appears to come from studies employing naturalistic training approaches, in which training activities are conducted using sport-specific stimuli or tasks [7]. Within this category of SVT, stroboscopic training has been the most extensively investigated strategy, with recent systematic reviews and meta-analyses reporting positive effects on sport-related outcomes (i.e., far transfer) [11–13]. Another naturalistic approach involves integrated training batteries, which combine multiple components (e.g., oculomotor and vergence drills, light-based reaction tasks, dual-task decision scenarios and agility under visual load) within structured protocols designed to challenge perceptual, cognitive and motor systems concurrently in sport-representative contexts [7]. However, the effectiveness of integrated SVT strategies remains debated. While some studies report improvements in trained component abilities, evidence for transfer to sport performance is mixed [10, 14,15]. Therefore, a meta-analytic approach can help estimate the overall efficacy of integrated SVT batteries on sport-relevant outcomes and identify moderators that influence their effectiveness.

The present study systematically reviews and meta-analyses the effects of integrated SVT batteries on time- and accuracy-dependent outcomes assessed during sport-specific tasks. Specifically, it aims to (1) quantify their effects on sport-specific performance outcomes, with particular emphasis on response accuracy and response time and (2) evaluate methodological quality and sources of heterogeneity across studies. This synthesis seeks to clarify whether multicomponent SVT interventions yield meaningful performance benefits and to inform evidence-based practice in sports vision and applied sports science. To enhance ecological validity, the analysis focuses on sport-relevant performance measures rather than visuo-cognitive laboratory metrics, which have uncertain translational value.

## Methods

**Fig. 1:**
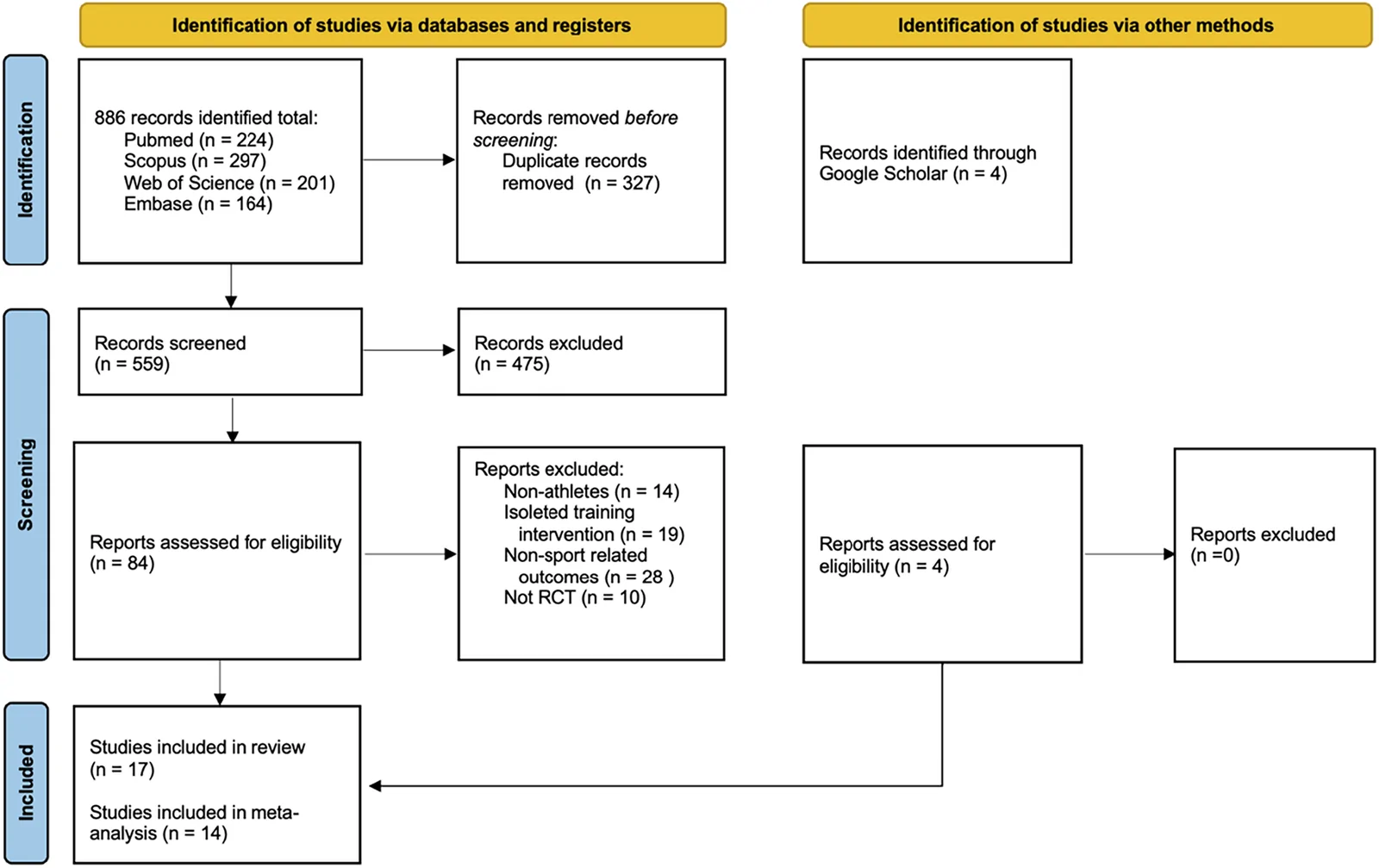
Preferred Reporting Items for Systematic Reviews and Meta-Analyses (PRISMA) flow diagram detailing inclusion and exclusion of studies. RCT randomised controlled trial.

This systematic review and meta-analysis were conducted in accordance with the Preferred Reporting Items for Systematic Reviews and Meta-Analyses (PRISMA 2020) guidelines [16]. The PRISMA flowchart for this study is illustrated in Fig. 1. The review protocol was prospectively registered in the International Prospective Register of Systematic Reviews (PROSPERO; registration number: CRD420251169720) on 16 October 2025.

### Information Sources and Search Strategy

The PubMed, Embase, Scopus and Web of Science Core Collection databases were searched systematically from inception to October 18, 2025. The search strategy combined terms related to sports vision and perceptual–cognitive training with descriptors for integrated, multicomponent or circuit-based interventions. In addition, reference lists of relevant reviews were screened manually to identify further eligible studies. The search strategy included the following terms: (“vis* training” OR “percept* training” OR “cogniti* training”) AND (“sport*” OR “athlet*” OR “on-field”), adapted for each database and applied to the title, abstract and keywords.

#### Study Selection and Eligibility Criteria

Study selection followed the PICO (Population, Intervention, Comparator, Outcome and Study design) framework to ensure methodological consistency and transparency. Studies were eligible for inclusion if they: (a) involved healthy participants engaged in athletic activities at the professional, amateur or recreational level; (b) investigated the effects of integrated visual–cognitive or perceptual–motor training batteries, defined as multicomponent programmes combining two or more visual, perceptual, cognitive and/or motor tasks delivered within a structured training protocol (e.g., circuit-based, immersive digital or cognitive–motor fusion formats); (c) reported at least one outcome related to sports performance, including on-field or validated sport-specific tasks and (d) employed an experimental, quasi-experimental or controlled pre–post design. Only peer-reviewed articles published in English were considered. Studies were excluded if they lacked a control or comparison group, involved non-athletic populations (e.g., clinical or paediatric samples), reported only laboratory-based visual, postural or biomechanical outcomes unrelated to sports performance or were published as reviews, meta-analyses, position statements or study protocols. Abstracts were not included because they often lack sufficient methodological detail to allow adequate assessment of risk of bias or applicability.

All identified records were imported into the Covidence systematic review software (Veritas Health Innovation, covidence.org), which automatically removed duplicates. Titles and abstracts were independently screened by two reviewers (JV and MC) and disagreements were resolved by consensus or, if necessary, by a third reviewer (BR). Full texts of potentially eligible studies were then assessed according to the predefined criteria by the same reviewers. Discrepancies were discussed and resolved by consensus, with arbitration by a third reviewer. In addition, backward (reference lists of included studies) and forward (citation tracking) searches were performed to identify additional eligible articles, which were evaluated using the same inclusion and exclusion criteria.

### Data Extraction

Two reviewers (JV and MC) independently extracted relevant information from the included studies, including bibliographic details, sport and participant characteristics, intervention components (e.g., drills, duration, frequency), comparator conditions and outcome measures. Summary statistics required for effect-size computation (means, standard deviations, sample sizes or test statistics) were recorded. Extracted data were entered into a predefined Microsoft Excel® spreadsheet (Microsoft.com) and any discrepancies were resolved through discussion or, if necessary, by a third reviewer (BR). Outcomes were categorised as time-dependent or accuracy-dependent and when multiple measures were reported, those most representative of on-field performance were prioritised. The studies of Steff et al. [17], Steff et al. [18] and Badau et al. [19] were identified as originating from the same experimental sample. To avoid duplication of data, only the first published report was included in the meta-analysis [17].

#### Methodological Quality Assessment

The methodological quality of included studies was evaluated using a modified *Downs and Black* checklist [20], which assesses reporting, external validity, internal validity (bias and confounding) and power. Consistent with prior sports science reviews [21,22], 17 of the 27 original items were retained as appropriate for the present designs. Each item was scored as “1” (yes) or “0” (no/unclear), yielding a maximum possible score of 17, with higher values indicating better reporting quality (Table S1). Quality assessments were conducted independently by two reviewers (JV and MC), with any disagreements resolved by consensus or, if necessary, by a third reviewer (BR).

#### Statistical analyses

Comparisons between experimental and control/placebo conditions were synthesised through meta-analytic procedures using *Review Manager* (RevMan, Version 5.0; Cochrane.org). Standardised mean differences (SMDs) were calculated for continuous outcomes to enable comparisons across studies employing different measurement scales. Effect sizes were interpreted in conjunction with corresponding *p*-values and 95% confidence intervals (CIs), as presented in the forest plots.

For quantitative analyses, sample size, mean and standard deviation values were extracted for both conditions from each study to compute the SMDs. When pre- to post-intervention comparisons were required and within-subject correlations were not reported, a conservative correlation coefficient of *r* = 0.5 was assumed. All analyses were conducted separately for time-dependent and accuracy-dependent outcomes.

Statistical significance was set at *p* < 0.05. Between-study heterogeneity was evaluated using Cochrane *Q*-statistics chi-square (Chi²) and the *I*², categorised as low (<25%), moderate (25–50%) or high (>50%) [23] Random-effects models (DerSimonian–Laird method) were applied a priori because variability among study designs, training protocols and participant characteristics was anticipated. Fixed-effects models were additionally used in sensitivity analyses when heterogeneity was low. Robustness of the pooled estimates was examined using a leave-one-out approach to identify the influence of individual studies on the overall results [24]. Potential publication bias and study influence were explored visually using funnel and Baujat plots [25,26].

## Results

### Study selection and characteristics

A total of 886 records were retrieved from the initial searches and four additional articles were found by backward and forward screening of the most relevant articles. After removing duplicates and screening titles and abstracts, 84 full-text articles were assessed for eligibility. In total, 17 studies were included in this systematic review and 14 in the meta-analysis. The study selection process is summarised in the PRISMA flow diagram (Fig. 1) and the characteristics of the included studies are presented in Table 1.

**Table 1:** Summary of studies assessing the long-term effects of integrated batteries of sports vision training.

Study	Participants (control/experimental)	Sport discipline	Training duration	Outcomes
Baccouch et al. [36]	24 young soccer players (12/12)	Soccer	4 weeks/16 sessions	Time-dependent
Balasaheb et al. [31]	20 male cricket batsmen (10/10)	Cricket	6 weeks/18 sessions	Accuracy-dependent
Bonato et al. [30]	20 male junior players (10/10)	Tennis	12 weeks/36 sessions	Accuracy-dependent Time-dependent
Campanella et al. [33]	27 elite judo athletes (14/13)	Judo	5 weeks/15 sessions	Accuracy-dependent
Guo et al. [29]	20 elite skeet shooters (10/10)	Skeet shooting	6 weeks/12 sessions	Accuracy-dependent
Hassan et al. [42]	24 junior male basketball players (12/12)	Basketball	10 weeks/40 sessions	Time-dependent
Hassan et al. [27]	24 junior male basketball players (12/12)	Basketball	10 weeks/40 sessions	Accuracy-dependent Time-dependent
Hassan [35]	28 female basketball players (14/14)	Basketball	10 weeks/40 sessions	Time-dependent
Hassan [28]	32 male amateur basketball players (16/16)	Basketball	8 weeks/32 sessions	Accuracy-dependent Time-dependent
Liu et al. [34]	14 male NCAA Division I baseball players (7/7)	Baseball	10 weeks/∼17 sessions	Accuracy-dependent Time-dependent
Lucia et al. [38]	24 young male semi-elite basketball players (12/12)	Basketball	5 weeks/10 sessions	Time-dependent
Lucia et al. [37]	52 young semi-elite basketball players (26/26)	Basketball	5 weeks/10 sessions	Time-dependent
Lucia et al. [40]	30 young elite basketball players (15/15)	Basketball	5 weeks/10 sessions	Time-dependent
Lucia et al. [39]	30 young skilled basketball players (15/15)	Basketball	5 weeks/10 sessions	Time-dependent
Paul et al. [32]	30 university level table tennis players (15/15)	Table tennis	8 weeks/24 sessions	Accuracy-dependent
Ramirez et al. [41]	32 male federated soccer players (18/14)	Soccer	8 weeks/24 sessions	Time-dependent
Steff et al. [17]	70 male junior basketball athletes (35/35)	Basketball	18 weeks/54 sessions	Time-dependent

*NCAA* National Collegiate Athletic Association (USA).

### Research Reporting Quality

The methodological reporting quality scores of the studies assessing the long-term effects of integrated batteries of SVT on outcomes related to sports performance were 14.9 ± 1.5 (mean ± SD) (Table S1, see supplementary material). Within this 17-point scale, scores around 15 indicate reasonably complete reporting but with important gaps. The most frequently missed items were question 24, related to the concealment of treatment allocation (adequately addressed in only one study) and question 27, concerning statistical power, with seven studies lacking sufficient power.

### Meta-analysis

#### Accuracy-dependent outcomes

An initial analysis including eight studies revealed substantial heterogeneity (Chi² = 54.88, *I*² = 89%, *p* < 0.001). The studies by Hassan [27,28] were subsequently excluded, as they contributed disproportionately to overall variability, reporting SMDs of −5.58 and −5.63, respectively.

**Fig. 2:**
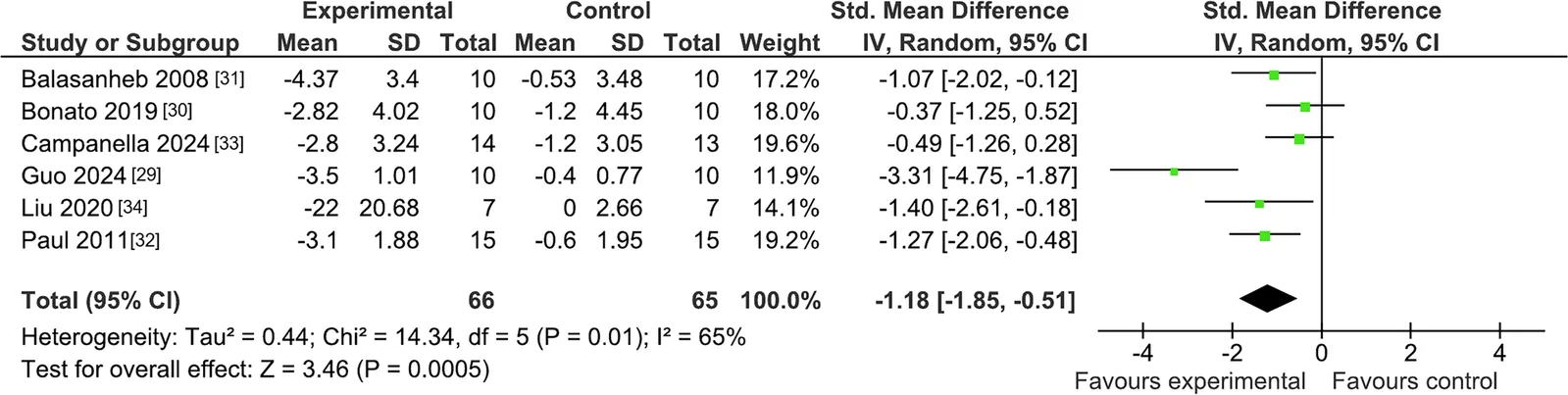
Forest plot demonstrating the acute effects of integrated batteries of sports vision training (SVT) on response accuracy. The forest plot shows standardised mean differences (SMDs) with 95% confidence intervals (CI) for six studies. The diamond at the bottom of the graph represents the pooled SMDs with 95% CI following fixed effects meta-analyses. The size of the plotted squares reflects the relative statistical weight for each study.

Consequently, a random-effects meta-analysis was conducted on six studies evaluating the effects of visual–cognitive training on accuracy-dependent measures of sports performance [29–34]. This analysis included a combined sample of 131 participants (66 in the experimental group and 65 in the control group). The pooled SMD was –1.18 (95% CI [–1.85, –0.51]), indicating a large overall improvement in accuracy for the experimental groups compared with controls (Fig. 2). Individual study effects varied considerably, with SMDs ranging from −0.37 [30] to −3.31 [29]. Moderate heterogeneity was observed (Chi² = 14.32, *I²* = 65%, *p* = 0.06) across studies.

The funnel plot (Fig. S1, panel A) showed a relatively symmetric distribution, with no extreme outliers detected. Visual inspection of the Baujat plot (Fig. S1, panel B) indicated that Guo [29] contributed most to overall heterogeneity, observing a significant reduction when this study was removed (Chi² = 4.04, *I²* = 1%, *p* = 0.40). The pooled SMD was reduced to –0.86 (95% CI [–1.26, –0.46]), with this effect being statistically significant. These results confirm that the observed improvement in accuracy is robust and statistically significant across all models, with only minor fluctuations in magnitude depending on the inclusion of individual studies.

#### Time-dependent outcomes

Thirteen studies met the inclusion criteria and were initially considered for quantitative synthesis. However, three studies were excluded from the final model due to their disproportionate contribution to overall heterogeneity and inconsistency in effect estimates [27,28, 35]. Specifically, these studies reported SMDs ranging from −13.59 to −34.70, values far exceeding the plausible range of effects observed across the remaining studies.

**Fig. 3:**
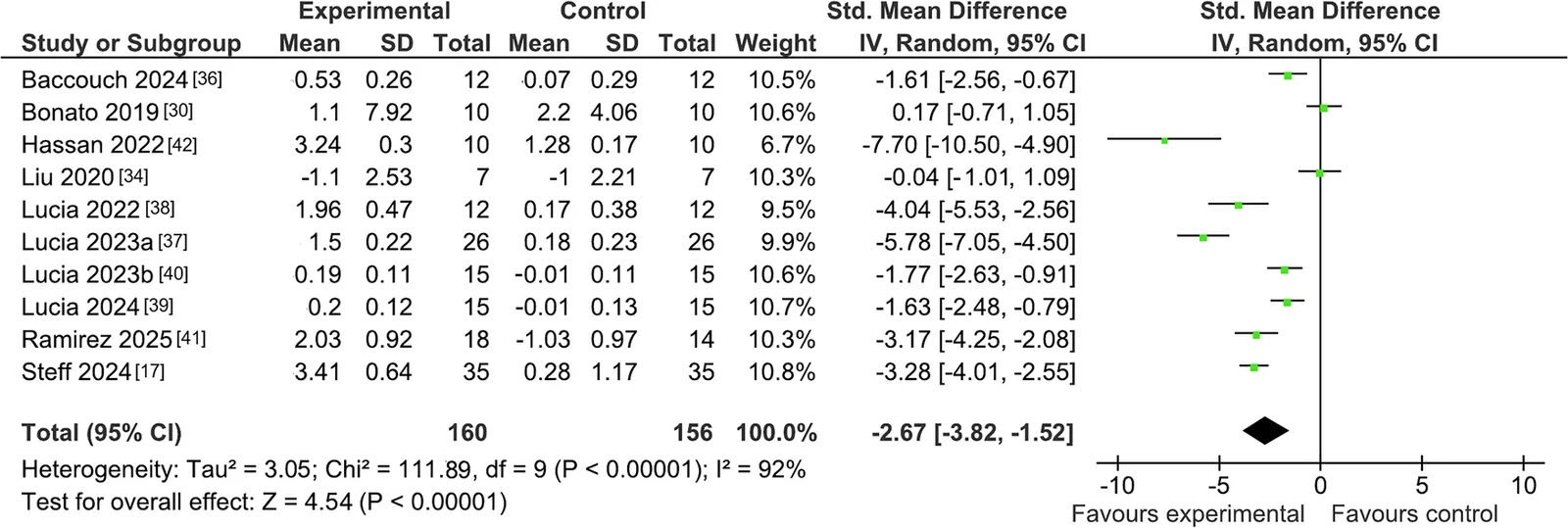
Forest plot demonstrating the acute effects of integrated batteries of sports vision training (SVT) on response time. The forest plot shows standardised mean differences (SMDs) with 95% confidence intervals (CI) for 10 studies. The diamond at the bottom of the graph represents the pooled SMDs with 95% CI following fixed effects meta-analyses. The size of the plotted squares reflects the relative statistical weight for each study.

The final meta-analysis included ten studies (*n* = 316 participants; 160 experimental, 156 control) [17, 30, 34, 36–42]. The pooled SMD across all studies was –2.67 (95% CI [–3.82, –1.52]), indicating a large and statistically significant improvement in response time following the intervention with integrated batteries of SVT (Fig. 3).

However, heterogeneity was substantial (Chi² = 111.89, *I²* = 92%, *p* < 0.001), suggesting that the observed outcomes varied considerably across studies. Visual inspection of the funnel and Baujat plots (Fig. S2) revealed a clear asymmetry, with the studies of Hassan et al. [42]. Bonato et al. [30] and Lucia et al. [37] showed the strongest influence on variability. Leave-one-out sensitivity analyses were conducted to further examine the influence of individual studies contributing most to residual heterogeneity. Specifically, removing Hassan et al. [42] slightly reduced the pooled effect to −2.30 (95% CI [–3.42, –1.18]) and heterogeneity remained stable (Chi² = 96.61, *I²* = 92%, *p* < 0.001). The exclusion of Bonato et al. [30] also caused minimal changes in SMD (−2.98 [−4.10, −1.85]) and heterogeneity (Chi² = 81.35, *I²* = 90%, *p* < 0.001). For its part, when Lucia et al. [37] was excluded, the pooled SMD showed a small decrease to −2.28 (95% CI [–3.34, –1.22]) and heterogeneity was very similar (Chi² = 78.74, *I²* = 90%, *p* < 0.001). Furthermore, exclusion of the three studies did not have a significant impact on the pooled effect (−2.17 [−3.09, −1.25]) or heterogeneity (Chi² = 39.09, *I²* = 85%, *p* < 0.001) [30, 37, 42]. These results reveal that the exclusion of these studies did not reduce heterogeneity significantly, nor alter the direction or statistical significance of the overall findings.

## Discussion

This meta-analysis provides the first quantitative synthesis of evidence examining the effects of integrated batteries of SVT on sport-specific performance outcomes. Across the included studies, these multicomponent interventions produced large and statistically significant improvements in both response time (SMD = −2.67, 95% CI [−3.82, −1.52]) and response accuracy (SMD = −1.18, 95% CI [−1.85, −0.51]) compared with control conditions. The findings indicate that training programmes targeting visual, cognitive and motor components simultaneously can meaningfully enhance time- and accuracy-dependent performance measures that are directly relevant to sport contexts. Importantly, despite the presence of heterogeneity, the direction and magnitude of effects remained robust across sensitivity analyses, underscoring the consistency of observed benefits across different sports, populations and methodological designs. Collectively, these results extend prior evidence on isolated visual or cognitive interventions by demonstrating that integrative, ecologically valid approaches are more likely to promote transferable improvements in sport-specific performance.

The consistent improvements in accuracy- and time-dependent outcomes likely reflect enhanced perception–action coupling promoted by integrated visual–cognitive training, which requires athletes to detect task-relevant cues, make rapid decisions and execute precise movements under realistic constraints [43,44]. This interpretation aligns with the Representative Learning Design framework, which emphasises the importance of preserving key informational variables from competitive environments to facilitate skill transfer [9]. By engaging perceptual, cognitive and motor processes simultaneously, integrated training batteries may also enhance attentional control and decision efficiency, thereby supporting faster and more accurate responses [45]. Together, these mechanisms help explain why ecologically valid, multicomponent interventions tend to produce larger and more transferable performance effects than isolated visual or cognitive drills.

The pooled effects observed in the present meta-analysis (SMD = −2.67 for time-dependent and −1.18 for accuracy-dependent performance) are notably larger than those reported in previous reviews examining isolated perceptual or cognitive training interventions. For example, Zhu et al. [46] reported a moderate improvement (SMD = 0.65) in real-game performance following perceptual-cognitive training, while Guo et al. [5] found a significant improvement in decision-making response time (SMD = 0.85) and sport-specific performance (SMD = 0.49) across different visual training paradigms. Recent meta-analytic evidence on stroboscopic training has also demonstrated significant improvements in various sport performance measures [11–13]. For instance, Vera and colleagues [13] reported effect sizes of −1.10 and −0.71 for time- and accuracy-dependent outcomes, respectively. These differences in effect magnitude likely reflect that interventions combining perceptual, cognitive and motor demands within representative sport contexts promote larger and more transferable performance gains than decontextualised drills [9, 44]. Collectively, these findings reinforce the view that multicomponent, representative training paradigms are particularly effective for eliciting meaningful and durable improvements in sport performance.

Substantial heterogeneity was observed in both analyses, particularly for time-dependent outcomes (*I*² = 92%) and, to a lesser extent, for accuracy-dependent measures (*I*² = 65%). This variability likely reflects differences across studies in training duration, intervention modality and participant characteristics. Comparable levels of heterogeneity have been reported in prior meta-analyses examining various SVT strategies [11, 13, 46], highlighting the methodological and conceptual diversity inherent to this research field. Importantly, sensitivity analyses demonstrated that the pooled effects were robust. Excluding individual studies with extreme SMDs or those contributing most to heterogeneity did not alter either the direction or statistical significance of the overall effects [27,28, 35] substantially. This consistency supports the reliability of the observed benefits of integrated visual–cognitive training despite methodological variability among studies. Although the inclusion of studies with diverse designs, control conditions and skill levels increases heterogeneity, it also enhances external validity by capturing a broad range of applied sport contexts. Future research would benefit from harmonising reporting standards and adopting more consistent training dosages, control interventions and outcome definitions to improve comparability across trials.

### Limitations and future research

The present meta-analysis has several limitations that should be acknowledged. First, although the pooled sample across studies was moderate, many individual investigations relied on relatively small sample sizes, which may inflate effect estimates and contribute to between-study heterogeneity. This limitation is common in applied sports science research and has been noted in previous systematic reviews of SVT [7, 47]. Future studies employing preregistered and adequately powered randomised controlled designs are needed to improve precision and generalisability. Second, there was considerable variability in the design and implementation of integrated training batteries, including differences in task composition, training volume, progression strategies and sport specificity. Although this heterogeneity likely reflects real-world practice and enhances ecological validity, it limits the ability to identify which specific components or task constraints are most critical for transfer. More detailed reporting of intervention characteristics and the adoption of standardised taxonomies for integrated training protocols would facilitate comparisons across studies and enable more informative moderator analyses [8, 15]. Third, most included studies assessed performance immediately after the intervention period, with limited follow-up data available. Consequently, the retention of training-induced gains remains unclear. This limitation mirrors concerns raised in previous reviews of both stroboscopic and perceptual–cognitive training, where retention effects are insufficiently documented [7, 12, 47]. Therefore, longitudinal studies examining retention, decay and potential booster effects represent an important direction for future research. Finally, relatively few studies included measures capable of elucidating the underlying mechanisms driving performance improvements, such as structural or functional brain changes. The inclusion of neurophysiological and neuroimaging outcomes would help identify the neural adaptations mediating these effects [48]. Future research should also focus on identifying the task constraints, training doses and contextual factors that maximise transfer [6,7, 14,15], as well as individual-difference moderators (e.g., expertise level, sport type and baseline perceptual–cognitive ability), to improve evidence-based prescriptions for applied practice.

### Practical applications

The findings of this meta-analysis have important implications for practitioners working in sports performance, including sport scientists and optometry specialists. The implementation of integrated batteries of SVT is associated with meaningful improvements in sport-specific performance, particularly in time- and accuracy-dependent outcomes that are central to competitive success. Notably, the magnitude of these effects is substantially larger than those reported for isolated SVT interventions (e.g., stroboscopic or quiet-eye training), indicating that integrated approaches may offer a meaningful performance advantage. Practitioners (i.e., coaches and optometrists) should prioritise training designs that preserve perception–action coupling, incorporate sport-representative stimuli and impose meaningful cognitive and temporal constraints. Programmes based on isolated visual drills or decontextualised exercises are unlikely to transfer effectively to on-field performance, consistent with critiques of component-based SVT [10, 45]. In contrast, integrated batteries that embed visual–cognitive challenges within realistic motor tasks align more closely with the informational demands of competition.

Additionally, the larger effects observed for time-dependent outcomes suggest that integrated SVT may be particularly beneficial in fast-paced sports where rapid decision-making and response execution are critical. Therefore, coaches and practitioners should consider aligning outcome measures and training goals with the temporal demands of the target sport. Given the variability in protocols across studies, practitioners are encouraged to individualise training content based on sport context, athlete level and performance needs, while adhering to principles of representativeness and progressive overload [8,9].

## Conclusions

This meta-analysis provides the first quantitative synthesis of evidence examining the effects of integrated batteries of SVT on sport-specific performance outcomes. The findings indicate that these multicomponent interventions produce meaningful improvements in both response time and response accuracy compared with control conditions. Despite substantial heterogeneity, the consistency and robustness of effects across sensitivity analyses support the conclusion that integrated SVT batteries can enhance performance in ecologically valid sport contexts. Collectively, these results extend prior work on isolated visual and perceptual–cognitive training by demonstrating that approaches integrating visual, cognitive and motor demands within representative tasks are more likely to promote far transfer. Future research should focus on refining these interventions, identifying optimal implementation strategies and establishing the durability of training-induced gains, thereby advancing evidence-based practice in sports vision and performance training.

## Supplementary information

Supplementary materials

## Data Availability

All data are available upon request from the corresponding author.
