# Supplementary material for: Effects of Integrated Sports Vision Training Batteries on Time- and Accuracy-Dependent Outcomes in Sport-Specific Tasks: A Systematic Review and Meta-Analysis
Source: Ophthalmic Physiol Opt. 2026 Jul 28;46(5):1006–13. doi: 10.1007/s44402-026-00147-8 (PMC13545032; doi:10.1007/s44402-026-00147-8)
Supplement: Supplementary file 1 [file 44402_2026_147_MOESM1_ESM.docx]

**SUPPLEMENTARY MATERIALS**

**Table S1.** Modified Downs and Black reporting outcomes.

|  | Item Number | | | | | | | | | | | | | | | | |  |
| --- | --- | --- | --- | --- | --- | --- | --- | --- | --- | --- | --- | --- | --- | --- | --- | --- | --- | --- |
| **Study** | 1 | 2 | 3 | 4 | 6 | 7 | 10 | 16 | 18 | 19 | 20 | 21 | 22 | 23 | 24 | 25 | 27 | **Total** |
|  |  |  |  |  |  |  |  |  |  |  |  |  |  |  |  |  |  |  |
| Baccouch et al. (2024) [36] | 1 | 1 | 1 | 1 | 1 | 1 | 1 | 1 | 1 | 1 | 1 | 1 | 1 | 1 | 0 | 1 | 1 | **16** |
| Balasaheb et al. (2008) [31] | 1 | 1 | 1 | 1 | 1 | 1 | 1 | 1 | 1 | 1 | 1 | 1 | 1 | 1 | 0 | 0 | 0 | **14** |
| Bonato et al. (2020) [30] | 1 | 1 | 1 | 1 | 1 | 1 | 1 | 1 | 1 | 1 | 1 | 1 | 1 | 1 | 1 | 1 | 0 | **16** |
| Campanella et al. (2024) [33] | 1 | 1 | 1 | 1 | 1 | 1 | 1 | 1 | 1 | 1 | 1 | 1 | 1 | 1 | 0 | 1 | 1 | **16** |
| Guo et al. (2024) [29] | 1 | 1 | 1 | 1 | 1 | 1 | 1 | 1 | 1 | 1 | 1 | 1 | 1 | 1 | 0 | 1 | 0 | **15** |
| Hassan et al. (2022) [42] | 1 | 1 | 0 | 1 | 1 | 1 | 1 | 1 | 0 | 1 | 1 | 1 | 1 | 1 | 0 | 0 | 0 | **12** |
| Hassan et al. (2023) [27] | 1 | 1 | 1 | 1 | 1 | 1 | 0 | 1 | 0 | 1 | 1 | 1 | 1 | 0 | 0 | 0 | 0 | **11** |
| Hassan et al. (2025a) [35] | 1 | 1 | 1 | 1 | 1 | 1 | 1 | 1 | 1 | 1 | 1 | 1 | 1 | 1 | 0 | 1 | 1 | **16** |
| Hassan et al. (2025b) [28] | 1 | 1 | 1 | 1 | 1 | 1 | 1 | 1 | 1 | 1 | 1 | 1 | 1 | 0 | 0 | 1 | 0 | **14** |
| Liu et al. (2020) [34] | 1 | 1 | 0 | 1 | 1 | 1 | 1 | 1 | 1 | 1 | 1 | 1 | 1 | 1 | 1 | 1 | 0 | **15** |
| Lucia et al. (2022) [38] | 1 | 1 | 1 | 1 | 1 | 1 | 1 | 1 | 1 | 1 | 1 | 1 | 1 | 1 | 0 | 1 | 1 | **16** |
| Lucia et al. (2023a) [37] | 1 | 1 | 1 | 1 | 1 | 1 | 1 | 1 | 1 | 1 | 1 | 1 | 1 | 1 | 0 | 1 | 1 | **16** |
| Lucia et al. (2023b) [40] | 1 | 1 | 1 | 1 | 1 | 1 | 1 | 1 | 1 | 1 | 1 | 1 | 1 | 1 | 0 | 1 | 1 | **16** |
| Lucia et al. (2024) [39] | 1 | 1 | 1 | 1 | 1 | 1 | 1 | 1 | 1 | 1 | 1 | 1 | 1 | 1 | 0 | 1 | 1 | **16** |
| Paul et al. (2011) [32] | 1 | 1 | 1 | 1 | 1 | 1 | 1 | 1 | 1 | 1 | 1 | 1 | 1 | 1 | 0 | 0 | 0 | **14** |
| Ramirez et al. (2025) [41] | 1 | 1 | 1 | 1 | 1 | 1 | 1 | 1 | 1 | 1 | 1 | 1 | 1 | 1 | 1 | 1 | 0 | **16** |
| Steff et al. (2024) [17] | 1 | 1 | 1 | 1 | 1 | 1 | 1 | 1 | 1 | 1 | 1 | 1 | 1 | 0 | 0 | 1 | 0 | **14** |

Note. Item numbers correspond to the modified Downs and Black checklist: 1 = hypothesis/aim/objective clearly described; 2 = main outcomes clearly described; 3 = participant characteristics clearly described; 4 = interventions clearly described; 6 = main findings clearly described; 7 = estimates of random variability provided; 10 = actual probability values reported; 16 = data dredging, if present, clearly reported; 18 = statistical tests appropriate for the data; 19 = compliance with interventions reliable; 20 = main outcome measures accurate, valid, and reliable; 21 = participants in different groups recruited from the same population; 22 = participants in different groups recruited over the same time period; 23 = randomization used to allocate participants; 24 = concealment of treatment allocation; 25 = adequate adjustment for confounding in the analyses; 27 = study had sufficient power to detect an effect.

**Figure S1**. **Accuracy-dependent outcomes**. **(A)** Funnel plot showing the relationship between standardized mean differences (SMDs) and their standard errors to assess potential publication bias or small-study effects. **(B)** Baujat plot illustrating the relative contribution of each study to overall heterogeneity (x-axis) and its influence on the pooled effect size (y-axis). The triangle in panel A represents the 95% confidence limits around the pooled SMD estimate.

**Figure S2. Time-dependent outcomes. (A)** Funnel plot showing the relationship between standardized mean differences (SMDs) and their standard errors to assess potential publication bias or small-study effects. **(B)** Baujat plot illustrating the relative contribution of each study to overall heterogeneity (x-axis) and its influence on the pooled effect size (y-axis). The triangle in panel A represents the 95% confidence limits around the pooled SMD estimate.
